# The Central Role of Cadherins in Gonad Development, Reproduction, and Fertility

**DOI:** 10.3390/ijms21218264

**Published:** 2020-11-04

**Authors:** Rafał P. Piprek, Malgorzata Kloc, Paulina Mizia, Jacek Z. Kubiak

**Affiliations:** 1 Department of Comparative Anatomy, Institute of Zoology and Biomedical Research, Jagiellonian University, Gronostajowa 9, 30-387 Krakow, Poland; paulinamizia@doctoral.uj.edu.pl; 2 The Houston Methodist Research Institute, Houston, TX 77030, USA; MKloc@houstonmethodist.org; 3 Department of Surgery, The Houston Methodist Hospital, Houston, TX 77030, USA; 4 MD Anderson Cancer Center, University of Texas, Houston, TX 77030, USA; 5 Cycle Group, Institute of Genetics and Development of Rennes, Faculty of Medicine, UnivRennes, UMR 6290 CNRS/UR1, F-35000 Rennes, France; 6 Department of Regenerative Medicine and Cell Biology, Military Institute of Hygiene and Epidemiology (WIHE), 01-163 Warsaw, Poland

**Keywords:** cadherin, cell adhesion, fertilization, folliculogenesis, gamete, germ cells, gonads, ovary, primordial germ cells, spermatogenesis, testis

## Abstract

Cadherins are a group of membrane proteins responsible for cell adhesion. They are crucial for cell sorting and recognition during the morphogenesis, but they also play many other roles such as assuring tissue integrity and resistance to stretching, mechanotransduction, cell signaling, regulation of cell proliferation, apoptosis, survival, carcinogenesis, etc. Within the cadherin superfamily, E- and N-cadherin have been especially well studied. They are involved in many aspects of sexual development and reproduction, such as germline development and gametogenesis, gonad development and functioning, and fertilization. E-cadherin is expressed in the primordial germ cells (PGCs) and also participates in PGC migration to the developing gonads where they become enclosed by the N-cadherin-expressing somatic cells. The differential expression of cadherins is also responsible for the establishment of the testis or ovary structure. In the adult testes, N-cadherin is responsible for the integrity of the seminiferous epithelium, regulation of sperm production, and the establishment of the blood–testis barrier. Sex hormones regulate the expression and turnover of N-cadherin influencing the course of spermatogenesis. In the adult ovaries, E- and N-cadherin assure the integrity of ovarian follicles and the formation of corpora lutea. Cadherins are expressed in the mature gametes and facilitate the capacitation of sperm in the female reproductive tract and gamete contact during fertilization. The germ cells and accompanying somatic cells express a series of different cadherins; however, their role in gonads and reproduction is still unknown. In this review, we show what is known and unknown about the role of cadherins in the germline and gonad development, and we suggest topics for future research.

## 1. Introduction

Normal gonad development assures the fertility and reproduction of an individual. Thus, the gonads are critical for the continuity of a species. The properly functioning gonads must possess a correct architecture, a sufficient number of viable germ cells, and assure the proper hormonal development and regulation of sexual features. The sexually differentiated gonad (testis or ovary) develops during the fetal life from the bipotential anlagen whose cells commit, during the process of sex determination, to divergent (female or male) fate. All these properties make the gonads an excellent model to study the molecular and cellular mechanisms involved in the tissue and organ specification and differentiation.

During morphogenesis, the proper cellular composition and structural organization of a developing organ result from the differentiation of cells into various tissue types, cell movement, and cell-specific gathering into groups. All these processes are regulated by the cytoskeleton and cell adhesion properties. Cell adhesion is a phenomenon that is critical for development, assuring the same cell type recognition and gathering. In 1939, the German–American developmental biologist Johannes Holtfreter coined the term “tissue affinity” to explain how the movements and selective adhesion of specific cell types are facilitated, resulting in the patterning of the tissues [1]. Later, in the 1980s, the Nobel Prize laureate Gerald Edelman postulated that the process of morphogenesis is regulated by molecules mediating cell adhesion. This indicated the key role of adhesion molecules in morphogenesis. In developing organs, different sets of these molecules at the surface of cells appeared to be involved in the specific sorting, migration, and organization into tissues, and therefore in the establishment of the organ identity and structure.

## 2. Cadherins—History and Function

Cadherins constitute the family of the most important group of adhesion molecules. The existence of this class of adhesion molecules has been proposed by Japanese embryologist Masatoshi Takeichi from Kyoto University in 1977. He reported a calcium-dependent (CD) cell surface protein while studying the adhesion of teratocarcinoma cells and fibroblasts [2]. Monoclonal antibodies (ECCD-1) recognizing this protein generated by his group allowed the target protein identification, isolation, and analysis by Western blotting [3]. The authors proposed calling this protein “cadherin” (originating from the “calcium-dependent adhesion”) [3]. Later studies showed that the non-epithelial tissues also contain cadherin-like molecules; therefore, they renamed the cadherin to E-cadherin (epithelial cadherin) and showed that it is expressed in various epithelial structures in the fetal and adult mouse [3]. The E-cadherin is also called cadherin 1 or Cdh1. Afterward, this group generated antibodies binding to the surface molecule of the neural cells, discovering the N-cadherin (neural cadherin or cadherin 2 or Cdh2) [4]. The same group also discovered the third cadherin, called the P-cadherin (placental cadherin or cadherin 3 or Cdh3) in the placenta [5], and the fourth cadherin, the R-cadherin (retinal cadherin or cadherin 4 or Cdh4) in the chicken retina [6]. The fifth cadherin, discovered by an Italian group in 1992 in the endothelium of blood vessels, was called the VE-cadherin (vascular endothelial cadherin or cadherin 5 or Cdh5) [7]. Although the original names of cadherins derived from the tissue in which they were discovered, further studies showed that they have a much broader expression. For example, it is now well documented that the E-cadherin expression is not limited to the epithelial cells.

**Figure 1:**
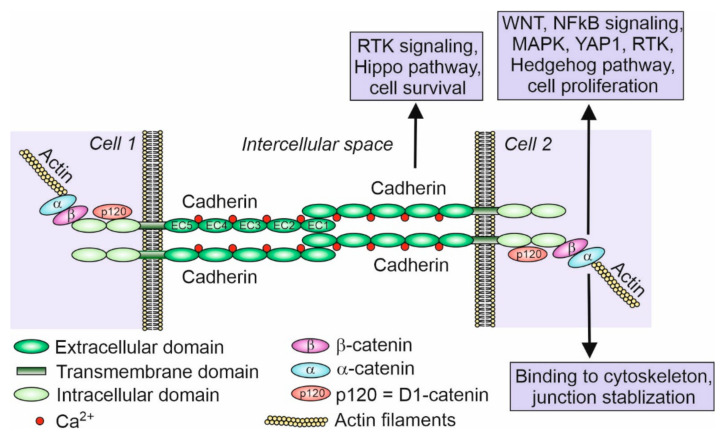
Scheme of classical cadherin binding between two cells. Classical cadherins, such as E- and N-cadherin, possess five extracellular domains (EC1–EC5), which bind Ca^2+^ ions, a transmembrane domain, and two intracellular domains. Intracellular domains bind p120 protein (D1-catenin) and β-catenin that binds α-catenin, which in turn binds actin filaments. Although there are many examples of cadherin and associated catenin signaling participating in various pathways (indicated in the figure within the boxes) such as cell proliferation and cell survival (inhibition of apoptosis) in different cell types, very little is known about the signaling and pathways regulated by cadherins in the developing and mature gonads [24,27,28,29,30]. Inspired by and modified from [25].

So far, 123 cadherins and cadherin-related genes have been discovered in chordates and grouped in several classes [8]. Cadherins and cadherin-related genes compose a superfamily of calcium-dependent transmembrane glycoproteins responsible for cell–cell adhesion or cell–cell recognition. They are defined by the presence of an extracellular domain (usually five or six or as many as 34) containing at least two calcium-binding cadherin repeats [9]. The signature for cadherins is at least two consecutive extracellular cadherin-specific motifs, which are called the cadherin repeats, with conserved calcium-binding amino acid residues [9]. Classical cadherins possess five extracellular domains (ECDs) which bind Ca^2+^ ions and cadherins of the adjacent cell, transmembrane domain, and two intracellular domains (Figure 1). Intracellular domains bind β-catenin that binds α-catenin, which in turn binds actin filaments.

Extracellular domains are directly responsible for the binding to the cadherins of the adjacent cell. The adhesive properties of cadherins are highly regulated at the molecular level. In the type I classical cadherins, the first ECD contains the HAV (His-Ala-Val) motif, whose role in adhesion has been studied [10,11,12]. Peptides containing the HAV sequence have been shown to block E-cadherin-mediated homotypic cell–cell adhesion of bovine brain microvessel endothelial cells [10]. This suggests the importance of the HAV site in the cadherin-mediated adhesion [11]. It has been also postulated that the HAV sequence is acting as the primary recognition site, and the flanking residues serve as regulators of the selectivity of the HAV region [12]. Moreover, not only the sequence or specific domains of cadherins are crucial for correct adhesion, but also the post-translational modifications and interactions with other proteins. *N*-glycosylation occurs at several asparagine residues of N-cadherin. In the *N*-glycosylation-deficient mutant cells, the N-cadherin becomes destabilized and degraded [13]. The glycosylation of N-cadherin modulates its interactions with other cadherins, which has an important impact on the intercellular binding [14]. The analysis of the role of *N*-glycosylation in male germ cells was performed in an elegant study of the *N*-acetylglucosaminyltransferase (GlcNAcT-I) MGAT1 that initiates the synthesis of complex *N*-glycans. The conditional deletion of *Mgat1* in spermatogonia (*Mgat1* cKO) leads to defects in the mouse spermatogenesis and severe infertility. This suggests that also the cadherins are possibly *N*-glycosylated by the MGAT1 in the adult male gonads. However, the loss of this post-translational modification of cadherins was not directly studied [15]. The E-cadherin is also robustly phosphorylated by specific kinases in the intracellular region that binds β-catenin. This phosphorylation may, depending on the cellular context, increase or decrease the adhesive activity of E-cadherin. The phosphorylation of three serines in this site promotes cell surface stability of E-cadherin and increases cell adhesion [16]. Cells with a hypophosphorylated E-cadherin exhibit lower cell–cell adhesion due to enhanced endocytosis and degradation of E-cadherin [16]. Mutations in the phosphorylation sites of E-cadherin prevent its binding to β-catenin and disrupt cell adhesion [17]. However, phosphorylation in other sites (such as Thr790) diminishes the interaction of E-cadherin with β-catenin and disrupts the homophilic interaction between the ECDs of E-cadherin [18]. The binding of E-cadherin to β-catenin depends not only on the phosphorylation but also the interactions with other factors. Numb protein and μ2 subunit of the AP-2 adaptor complex are the best-described examples. These cadherin-associated proteins enhance the endocytosis of cadherins decreasing cell adhesion. For example, Numb facilitates the endocytosis of E-cadherin by interacting with its binding partner p120 catenin [19]. This indicates that the cell adhesion may be decreased by the internalization of cadherins through clathrin-mediated endocytosis (CME). The μ2 subunit of the AP-2 adaptor complex interacts with several motifs of the intracellular domain of N-cadherin, and it is responsible for its endocytosis [20], while the β-catenin inhibits N-cadherin endocytosis by masking these motifs. The removal of β-catenin enables μ2 binding to N-cadherin, thereby increasing clathrin-mediated N-cadherin endocytosis in the neurite outgrowth. Although mentioned above, molecular mechanisms regulating cadherins function and impacting cell adhesion have been described for several systems, but it is plausible that the same mechanisms operate during gonad development.

Cadherins play important roles in developmental processes, especially in cell sorting and the formation of tissues, which are guided by the homotypic interactions of cells with the same set of cadherins at their surfaces [21]. Cadherins contribute to tissue integrity and resistance to stretching [22]. Responding to the physical forces, cadherins participate in the mechanotransduction. Cadherins also participate in carcinogenesis and are important targets for novel anticancer therapies [23]. Cadherins along with associated catenins also act as receptors for signaling molecules and are involved in the mediation of several signaling pathways (Figure 1). This makes them the key factors in the signal transduction between cells. By capturing signals from extracellular space or adjacent cells, cadherins are involved in the regulation of cell proliferation, apoptosis, and cell differentiation [24,25]. The E-cadherin has been shown to suppress Wnt/β-catenin signaling and the receptor tyrosine kinases/phosphatidylinositol-3-kinase (RTK/PI3K) pathway in epithelial cells [26]. The N-cadherin mediates the activation of mitogen-activated protein kinase (MAPK)/extracellular signal-regulated kinases (ERK) to enhance cell survival and the migration of non-epithelial cells [26]. E- and N-cadherin along with associated catenins have also been shown to participate in NF-κB-mediated signaling, RhoA GTPase signaling, and the Hippo, YAP1, and RTK and Hedgehog pathways [25,27,28,29,30,31]. Among these cell-signaling pathways, the Wnt4/β-catenin signaling is key for female sex determination and ovary differentiation in mouse and other vertebrates [32]. Although it can be assumed that cadherins also participate in the regulation of gonad development by the mediation of cell signaling, so far, there is no information on this issue.

## 3. Members of the Cadherin Superfamily

The cadherin superfamily is divided into three groups: (I) 30 classical cadherins, (II) 76 protocadherins, and (III) 17 cadherin-related proteins (Table 1).

Although the majority of the studies on the role of cadherins in gonad development have focused on E- and N-cadherin, and there are only a few studies on VE- and P-cadherins; meanwhile, the global analysis of developing mouse gonad transcriptomes revealed the expression of many other cadherins and protocadherins [33]. Table 2 summarizes the expression pattern and distribution of cadherins in the developing mouse gonads. Various cadherins expressed in the somatic and germ cells of the gonads are the focus of this review.

## 4. Cadherins and the Primordial Germ Cells (PGCs)

**Figure 2:**
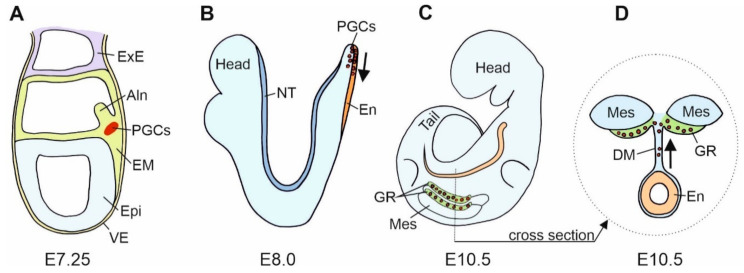
Scheme of the mouse primordial germ cells (PGCs) fate. (**A**). In the mouse gastrula at E7.25, E-cadherin positive (+) PGCs are present at the site of their origin, the E-cadherin positive embryonic mesoderm (EM). At this stage, mesodermal cells segregate into a group of E-cadherin positive somatic cells, E-cadherin positive PGCs, and E-cadherin negative (−) cells that form the allantois (Aln). (**B**). In the mouse neurula at E8.0, PGCs translocate from the mesoderm to the E-cadherin positive endoderm, En (a region of hindgut formation). At this stage, the expression of E-cadherin in PGCs decreases. Subsequently, PGCs migrate individually in the anterior direction. (**C**) (lateral view) and (**D**) (cross section)**.** In the mouse embryo at E10.5, PGCs leave the hindgut, increase E-cadherin expression and interconnect by filopodia. PGCs migrate dorsally through dorsal mesentery (DM) to the genital ridges (GR). Epi—epiblast; ExE—extraembryonic ectoderm; Mes—mesonephros; NT—neural tube; VE—visceral endoderm.

Gonads are built of the germ cells and scaffolding somatic cells. These two different cell lines originate in the different locations in the embryo. They meet and aggregate within the genital ridges that are the earliest gonadal anlage. The earliest stage of the germline formation is the stage of primordial germ cells (PGCs). PGCs derive from remote regions such as the vegetal pole of an early anuran embryo or the extraembryonic regions, such as the germinal crescent of a chicken embryo [34]. The differentiation of embryonic cells into PGCs is determined by two mechanisms: (i) the cytoplasmic determinants deposited in the ooplasm during oogenesis, e.g., anuran amphibian, *Xenopus laevis*, or (ii) the induction (signaling) from the adjacent cells, e.g., urodele amphibians, such as axolotls and mammals. PGCs are pluripotent, and the repressed RNApolII-dependent transcription prevents their somatic differentiation in favor of the germline fate maintenance [35]. In mice, PGCs segregate from the pluripotent cell population of the most proximal epiblast after the onset of gastrulation at embryonic day 6 (E6), leave the proximal epiblast between E6.5 and E6.75, and reach the proximal region of extraembryonic mesoderm (at the base of the incipient allantois) at E7.25 [36] (Figure 2A).

The PGCs and the precursors of PGCs express the E-cadherin. In mice, cells of the proximal region of extra-embryonic mesoderm, from which the PGCs derive, express E-cadherin [37]. Blocking E-cadherin with the ECCD-1 antibodies in vitro inhibits PGC formation [37]. This indicates that the E-cadherin-mediated cell–cell interactions are necessary for PGCs’ commitment to the germ cell fate [37]. These authors postulate that the E-cadherin plays a key role in the clustering of the PGC precursors before they become committed to the PGC fate and that the PGC specification occurs through the E-cadherin-mediated cell–cell interaction between the PGCs precursors, but not between the precursors and neighboring somatic cells [37]. There are several possible explanations for the mechanisms involved. The E-cadherin may simply transmit the instructive signals for the PGC determination or it may allow close contact for the transmission of juxtacrine (contact-dependent) signals. Alternatively, E-cadherin may anchor the PGCs precursor within the PGC differentiation niche [37]. Another possibility is that the homotypic interactions between PGCs and the cells of the proximal region of extraembryonic mesoderm, both expressing the E-cadherin, may prevent PGC precursors from moving to the distal region of the mesoderm, where the differentiation of allantois takes place, and it thus prevents the differentiation of PGCs into other cell lines, such as the allantois [37] (Figure 2A).

## 5. Cadherins and PGCs Migration

The PGCs actively migrate from the place of their origin to the genital ridges. In the mouse, this process takes 4 days. In the mouse and the African clawed frog, *Xenopus laevis*, the PGCs migrate first anteriorly, through the endoderm, along the gut, and then dorsally through the dorsal mesentery of the gut, to the genital ridges forming at the ventral surfaces of the mesonephroi (embryonic kidneys) [38] (Figure 2B–D). In chicken, the PGCs migrate within the blood vessels from the extraembryonic germinal crescent to the genital ridges [38]. In zebrafish, the PGCs first locate at four sites at the dorsal part of the embryo and then migrate, in two groups, through the lateral mesoderm toward the genital ridges [39]. Throughout their migration, PGCs form contacts with the adjacent cells, which direct their migration. After arrival to the genital ridges, the PGCs acquire contact with the somatic cells of the genital ridges, which, in turn, switch off the PGCs’ migration program. Initially, it was reported that the PGCs migrate in groups of cells interlinked by the network of cytoplasmic processes [40]. Studies of Di Carlo and De Felici (2000) showed that the migrating PGCs isolated from mouse embryos, and the germ cells isolated from the gonads, rapidly aggregated in vitro in the presence of Ca2+ ions, and the aggregation was inhibited by the ECCD-1 antibody-supplemented medium [41]. Disrupting E-cadherin caused problems with PGC–PGC contacts and caused PGCs to be lost outside the gonad. This proved the role of E-cadherin in PGC migration.

One of the first attempts to evaluate the exact role of E-cadherin in PGCs migration was performed by culturing the slices of mice embryos at E10.5 in the medium supplemented with anti-E-cadherin antibodies [42]. Although the inhibition of E-cadherin did not eliminate PGC migration in a proper direction, it inhibited their aggregation into the groups within the genital ridges. This indicated that E-cadherin is not required for PGCs migration per se but rather aggregation [42]. The expression of E-cadherin changes during the migration. When the PGCs leave the place of origin (the proximal extraembryonic mesoderm), they express a high level of E-cadherin. The level of E-cadherin expression decreases during the active PGCs migration through the endoderm in the hindgut. A decline in E-cadherin expression leads to PGCs individualization and dispersion, and it lowers their affinity to the surrounding endodermal cells (which express a high level of E-cadherin) facilitating migration [43]. Such a transient decline of E-cadherin expression in the migrating PGCs was also reported in zebrafish [44] and *Xenopus* [45]. Kunwar et al. (2008) showed that in *Drosophila*, a decrease in the DE-cadherin (*Drosophila* version of E-cadherin) in PGCs leads to the premature PGC dispersal [46]. These studies indicate that the downregulation of E-cadherin during PGC migration is crucial for their dispersion. Studies in zebrafish also showed that a lower expression of E-cadherin in migrating PGCs is necessary for the formation of cell protrusions and contacts with adjacent somatic cells to generate an appropriate traction force during the migration [47]. The lower level of E-cadherin allows the faster turnover of contacts and increases migration speed [48]. In contrast, the over-expression of E-cadherin leads to the increased formation of the PGC protrusions and dramatically reduces migration speed with some cells extending protrusions in all directions and others becoming immobile. This suggested that a strictly controlled E-cadherin expression level is important for the optimal migration speed rather than cell guidance [48].

After the PGCs migrated individually through the endoderm, they leave the gut and migrate, as a group of filopodia-connected cells, through the dorsal mesentery toward the genital ridges (Figure 2B–D). During the migration of PGCs in the mesentery, they again upregulate the expression of E-cadherin, which leads to the formation of the inter-PGC contacts [42].

A mechanism regulating the E-cadherin expression in migrating PGCs is not clear. So far, two genes have been described as being involved in this process. In zebrafish, the dead end (dnd) gene that encodes an RNA-binding factor is required for the decrease of E-cadherin expression at the beginning of PGC migration. The dnd knockout leads to the maintenance of the high level of E-cadherin, resulting in the non-dispersing PGCs with multiple adhesion contacts [44], while the Rgs14a (Regulator of G-protein signaling 14) signaling factor inhibits the decrease of E-cadherin [49]. The lack of Rgs14a causes a premature decrease in E-cadherin expression, and its overexpression inhibits E-cadherin decrease [49]. In chicken, the ectopic retinoic acid (a derivative of the vitamin A) increases the expression of E-cadherin and enhances PGCs aggregation. The PKC (protein kinase C) inhibits retinoic acid effects on E-cadherin expression [50].

However, not only the level of E-cadherin expression is important for the migration of PGCs, but also the localization of E-cadherin within the PGCs. In *Drosophila*, E-cadherin moves to the posterior tip of the polarized migrating PGCs. In zebrafish, the PGCs use the retrograde flow of the cytoplasm on the cytoskeleton to generate E-cadherin-mediated forces that provide traction between PGCs and the surrounding tissues [47]. In addition to the E-cadherin, also the P-cadherin is expressed in the migration and settled in the genital ridges of the PGCs [42]. N-cadherin is absent from the migrating PGCs and appears later when the PGCs are already in the gonads.

## 6. Cadherins in Gonad Development

After arriving at the genital ridges, the PGCs aggregate, lose the ability to migrate, and become enclosed by the somatic cells of genital ridges. The somatic cells derive from the proliferating coelomic epithelium covering the early gonad [38]. The PGCs located within the genital ridge are called the germ cells or gonocytes [51]. A subsequent proliferation of coelomic epithelium and germ cells leads to the growth of, still sexually undifferentiated, gonads.

In mice, sexual differentiation occurs in the embryo between E10.5 and E12.5 [38]. The differentiating testes develop the testis cords containing germ cells surrounded by the somatic cells that differentiate into Sertoli cells. Testis cords are embedded in the interstitium that becomes invaded by the mesonephros-derived cells, which form the blood vessels. Some of the interstitial cells differentiate into steroidogenic fetal Leydig cells. Postnatally, the testis cords acquire the lumen and become the seminiferous tubules.

The differentiating mouse ovaries contain the germ cell cysts built of groups of germ cells surrounded by the somatic cells. Around stage E14.5, the germ cells enter meiosis, and the germ cell cysts brakes into ovarian follicles, which contain single oocytes surrounded by the layer of follicular cells (the ontogenic equivalent of the Sertoli cells in the testes). Ovarian follicles are embedded in ovarian stroma, which is a homolog of the testis interstitium. The stroma around the follicles differentiates into steroidogenic theca cells [38]. Thus, the developing gonads, both testes and ovaries, are composed of a mixture of several cell lines originating from different sources. These cell lines differ, among many other properties, in the expression of various cadherins, which are crucial for the formation of the gonad structure.

E- and N-cadherin are critical for the gonad structure. The somatic cells of genital ridges express N-cadherin, and the PGCs express E-cadherin. In mice, starting at stage E10.5 until sexual differentiation, the germ cells upregulate the expression of E-cadherin, allowing their aggregation in the gonads. This expression of E-cadherin in the germ cells is maintained during the further development of gonads [42,52]. When all germ cells are already settled in the genital ridges (about stage E12.5), the germ cells also start to express the N-cadherin, which probably enhances the adhesion with the somatic cells [42].

**Figure 3:**
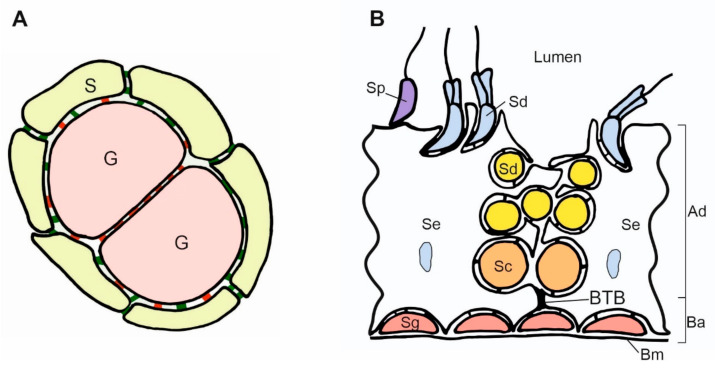
Scheme of contacts between germ and somatic cells in the gonads. (**A**). Adjacent germ cells (G) in the nest are connected by E-cadherin (red) and enclosed by the somatic cells (S). The N-cadherin (green) is present between somatic cells, and both E- and N-cadherin are present between somatic and germ cells. (**B**). Scheme of the mouse seminiferous epithelium. The spermatogonia (Sg) are located in the basal region of the seminiferous epithelium and adhere to the basement membrane (Bm) and Sertoli cells (Se). The accumulation of the adherence junctions forms the blood–testis barrier (BTB) above the spermatogonia. BTB divides the epithelium into two compartments: basal (Ba) and adluminal (Ad). The transitional disassembly of BTB allows pachytene spermatocyte (Sc) passage from the basal to the adluminal compartment. While spermatogenesis progresses, the round and elongated spermatids (Sd) become oriented toward the lumen of the seminiferous tubules. All the germ cells form contacts with Sertoli cells. Spermatids develop flagellum while still attached to the Sertoli cells. In the process of spermiation, the germ cells become released (detached) from the Sertoli cells, and from that moment, they are called the spermatozoa (Sp). The spermatogenesis, including BTB assembly and disassembly, the passage of germ cells toward the lumen, and spermatozoa release, are regulated by changes in cell adhesion.

Fleming and colleagues (2012) showed the details of the localization of N- and E-cadherin in developing mouse gonads using immunofluorescence [53]. The expression of E-cadherin prevails in the germ cells, while the expression of N-cadherin prevails in the somatic cells. The somatic–somatic cell contacts are positive for N-cadherin only, and the germ–germ cell contacts are positive for E-cadherin only (Figure 3A). However, the contacts between somatic and germ cells are positive for both N- and E-cadherin, with a strong E-cadherin signal and a low N-cadherin signal [53]. Bendel-Stenzel and colleagues (2000) showed that the E-cadherin is distributed along the germ cell membrane with the increased signal at the germ–germ cell contacts [42]. In contrast, the N-cadherin localized focally at the germ cell membrane [42]. Thus, the E-cadherin facilitates the germ cell clustering, while the N-cadherin together with E-cadherin facilitates the germ–somatic cell interactions.

The sexually differentiating gonads show the sex-specific differences in cadherin expression. Somatic cells in the XY mouse gonads (differentiating testes) show higher expression of N-cadherin compared to the XX gonads (differentiating ovaries) [53]. This may explain why the male germ cells become enclosed by a tight layer of pre-Sertoli cells (which express a high level of N-cadherin). Such sex-specific expression of cadherins presumably is one of the key factors driving the sexual differentiation of gonads.

Cadherins also seem to participate in the compartmentalization of the internal structure of the gonads. In the differentiating mouse testis, the Sertoli cells, in contrast to the interstitium, maintain a high expression of N-cadherin [53]. Such a differential expression of N-cadherin decreases the affinity between Sertoli and interstitial cells and triggers the separation of these two cell lines into two different structures (cords and interstitium, respectively). In already sexually differentiated mouse testes, the expression of E-cadherin in germ cells decreases at E15.5, i.e., soon before birth [41]. In the testes, this specific decrease in E-cadherin expression in the germ cells depends on the Dmrt1 (doublesex and mab-3-related transcription factor) gene that controls testis development. The deletion of Dmrt1 in the germ cells leads to the high expression of E-cadherin and the maintenance of germ cell stemness (pluripotency) [54]. It is unknown how the elevated E-cadherin expression allows maintaining the stemness. In addition, in *Drosophila*, E-cadherin is involved in the maintenance of germ cells in the stem cell niche [55,56].

In developing mouse ovaries, the oogonia in the germ cell cysts and the early oocytes in the ovarian follicles express E-cadherin. However, soon after, the expression of E-cadherin in the oocytes decreases [41]. In the developing ovaries, during the cyst breakdown, specific changes in E-cadherin expression occur. In mice, E-cadherin is initially intensively located at oocyte–oocyte contacts until E17.5, and after that time, the localization of E-cadherin becomes weaker and diffused [57]. The expression of E-cadherin again increases by P4. Between E17.5 and P4, ovarian cyst breakdown occurs, and the transitional decrease in E-cadherin expression in this period possibly allows oocyte separation in the process of cyst breakdown. It was also observed in the hamster ovaries [58]. This E-cadherin decrease is an effect of JNK signaling. The in vitro inhibition of this signaling pathway enhanced the expression of E-cadherin and disrupted cyst breakdown [57]. On the other hand, the in vitro inhibition of E-cadherin accelerated cyst breakdown and primordial follicle formation.

The expression of E-cadherin in the oocytes has been proven in human, mouse, and rat oocytes. It is the highest in the oocytes in the fetal ovaries and then once again in the neonatal ovaries, and later, it declines again. In the rat, the E-cadherin expression peaks at the 7th postnatal day and then declines [59]. This indicates that E-cadherin may be especially important at the early stages of folliculogenesis that occur in the prepubertal period. Afterward, E-cadherin is expressed at all stages of folliculogenesis. In the ovarian follicles, E-cadherin colocalizes with N-cadherin, which suggests their heterotypic interaction at the boundary of follicular cells and oocytes [60]. E-cadherin is also expressed in the interstitial (stromal) cells between the ovarian follicles, including theca cells enclosing the follicles [61].

In the perinatal period, a non-renewable pool of primordial follicles is established. E-cadherin was proved to be crucial for establishing this pool, since the loss of E-cadherin both due to the genetic knockout in vivo and in vitro knockdown leads to the loss of this pool due to increased oocyte apoptosis [62,63].

Functional analysis of the role of E- and N-cadherin in the development of mouse gonads has been difficult because of embryonic lethality. The mouse embryos with a mutation in E-cadherin or N-cadherin die at the 32-cell stage and E10 stage, respectively [64,65]. To overcome the lethality, we performed conditional knockouts of E- or N-cadherin in the germ cells or somatic cells of developing mouse gonads [62,66].

The knockout of E-cadherin in the germ cells or in the somatic cells led to the apoptotic germ cell loss both in developing testes and ovaries [62] but did not affect the somatic cells of the gonads; the testis cords (although sterile) and the interstitium differentiated normally. This indicates the critical role of E-cadherin for the survival of the germ cells in developing gonads. This suggests that the mutations in E-cadherin may be responsible for infertility in humans. In contrast, the E-cadherin is less, or not at all, important for the adhesion between the somatic cells of developing gonads and the gonad architecture. This is consistent with the fact that the somatic cells express much less E-cadherin than the germ cells [33].

The knockout of N-cadherin in the germ cells led to apoptosis and a reduction of germ cell number [66]. The knockout of N-cadherin in gonadal somatic cells, in contrast to the E-cadherin knockout, disrupted the gonad structure. The lack of N-cadherin in somatic cells led to their dispersion, the malformation of the testis cord, and aberrant interstitium with a lower number of steroidogenic fetal Leydig cells. This indicates that N-cadherin is critical for the adhesion of somatic–somatic cells and in the establishing of the gonadal structure.

P-cadherin (placental cadherin, Cdh3) is another cadherin expressed in developing gonads [33,67]. It is expressed in all somatic and germ cells in both sexes, with higher expression in the germ cells and Sertoli cells in differentiating testes [33]. The role of P-cadherin in gonad development remains unknown. The mice with P-cadherin knockout are viable and fertile, which suggests that the presence of P-cadherin is not critical for germ cells and gonads development [66]. It has been proposed that although the P-cadherin has a role in the adhesion between germ cells and germ and somatic cells, its absence can be compensated for by other intercellular adhesion molecules [42].

VE-cadherin (vascular endothelial cadherin, Cdh5) is another cadherin participating in the testis development. In XY mice, after stage E11.5, the mesonephros-derived VE-cadherin positive cells invade the differentiating testes [68]. These cells give rise to a testis-specific vasculature that is critical for the patterning of the testis structure. The anti-VE-cadherin antibody added to the mouse XY gonad culture medium disrupts migration of the mesonephros-derived cells to the testes and inhibits vasculature and testes cord formation [68]. Thus, VE-cadherin is critical for the testis structure development by mediating vascularization.

## 7. Cadherins in Adult Testis

The adult testis is composed of the seminiferous tubules containing the germ and Sertoli cells, the peritubular cells covering the seminiferous tubules, the interstitium containing the testosterone-producing Leydig cells, and the blood vessels. Cadherins-mediated cell contacts maintain the integrity of seminiferous epithelium and the general structure of the gonad. Contacts between cells in the seminiferous epithelium are also crucial for the production of the mature sperm (Figure 3B). N-cadherin is expressed by Sertoli and germ cells in the adult testes; it promotes their adhesion, which can be blocked by N-cadherin antibodies, and it plays a pivotal role in spermatogenesis [69]. The Leydig cells and peritubular cells surrounding the seminiferous tubules are N-cadherin-negative [70,71]. In the interstitium, only the endothelial cells are N-cadherin-positive. In human testes, the N-cadherin is localized at the surface of Sertoli cells, between Sertoli cells and spermatogonia, spermatocytes, and early spermatids; however, late spermatids and spermatozoa are negative for N-cadherin. In mouse testes, the N-cadherin is also present in the late spermatids, while in rats, the N-cadherin was detected only in the spermatogonia located in the lower part of seminiferous tubules [70,71]. This may indicate different mechanisms controlling spermatogenesis in different species.

The seminiferous tubules contain a specific complex of cell adhesion molecules that tightly close the space between adjacent Sertoli cells. This allows the formation of the isolated niche for the development of spermatogonia and creates the so-called blood–testis barrier (BTB) (Figure 3B). The N-cadherin was shown to be an important component of cell adhesion complexes (adhesion junctions) in BTB [72]. Moreover, N-cadherin is a component of the ectoplasmic specializations—the testis-specific anchoring junctions are responsible for the adhesion of germ cells to the Sertoli cells.

During spermatogenesis, the germ cells at consecutive stages, the spermatogonia, spermatocytes, and spermatids pass between adjacent Sertoli cells toward the seminiferous tubule lumen. This movement is driven by changes in the adhesion between the Sertoli–Sertoli cells, germ–germ cells, and Sertoli–germ cells. The dynamic changes in the adhesion include the turnover of cadherins (endocytosis followed by recycling back to the cell surface) [73]. The endocytosis of N-cadherin causes the disintegration of adherens junctions in BTB, and the spermatocytes can move to the adluminal compartment of the seminiferous tubule. Subsequently, the N-cadherin is recycled back to the cell membrane, and the BTB is restored. The N-cadherin dynamic allows a strict temporal and special control of spermatogenesis.

The expression of N-cadherin in the testis is hormonally regulated. The testosterone enhances the endocytosis and recycling of N-cadherin [63]. Testosterone and FSH (follicle-stimulating hormone) significantly increase N-cadherin expression in Sertoli cells of rat and enhance adhesion between the round spermatids and Sertoli cells in vitro, which can be blocked by the anti-N-cadherin antibodies [74]. This explains why the low level of testosterone induces the detachment of round spermatids from Sertoli cells and prevents the formation of elongated spermatids.

The downregulation of N-cadherin expression also allows releasing the spermatids to the lumen of tubules (spermiation) in mice [75]. Early elongated spermatids express a high level of N-cadherin, while the late elongated spermatids soon before spermiation show low N-cadherin expression. The downregulation of N-cadherin loosens the adhesion between Sertoli cells and spermatids, which results in the release of the spermatids to the lumen of the seminiferous tubule (Figure 3B). The knockout of N-cadherin in adult mouse Sertoli cells does not affect the integrity of seminiferous epithelium but disrupts spermatogenesis. The germ cells translocated from the peripheral to the adluminal region, and the number of germ cells decreased due to enhanced apoptosis [76].

The role and expression of E-cadherin in the testes are more controversial. E-cadherin is highly expressed in the fetal gonads and is also detected in immature testes before the formation of the BTB. Although the early studies reported the absence of E-cadherin expression in the adult testis [70,77], more recent studies showed the presence of E-cadherin in undifferentiated spermatogonia [78,79], and the earliest spermatogonial cells (SSCs or spermatogonial stem cells). The E-cadherin expression decreases in the late spermatogonia [80].

The expression of P-cadherin was also studied in the testes. P-cadherin was found in the mouse Sertoli cells in the postnatal period and was lost after puberty. P-cadherin is also expressed in adult peritubular myoid cells but not in the postnatal period [52]. The adult testes express seven classical cadherins (E-, N-, P-cadherin, cadherin-6, -8, -10, and -11), 14 protocadherins (protocadherin 6, A3, A4, A8, A9, A10, A11, C3, T1, T2, T3, T4, T5, and T6), and 4 cadherin-related genes (Flamingo 1a and 1b, FAT and VE-cadherin 2) [81].

## 8. Cadherins in Adult Ovary

The postnatal ovaries contain the primordial follicles, in which the prophase-arrested oocytes are enclosed by a monolayer of flattened pre-follicular cells [82]. Due to an increase of follicular cell layers around the oocyte, the primordial follicles in adult ovaries transform into primary and secondary follicles followed by the Graafian follicles, which during ovulation, rapture and release the cumulus–oocyte complex (COC) [82].

In adult mouse ovaries, E-cadherin is strongly expressed in the oocytes but not in the follicular cells [60]. E-cadherin shows a punctate pattern of localization at the membrane of the oocyte at all stages of follicle development. In contrast, the N-cadherin protein and mRNA are barely detectable in oocytes but are present in the follicular cells at all stages of follicle development [60]. N-cadherin is expressed in the follicular cells in the primordial and primary follicles and highly expressed in the antral follicles in all layers of follicular cells [83]. In the atretic follicles, N-cadherin is expressed in the outer layers of follicular cells. N-cadherin is critical for the survival of the follicular cells in the ovaries. Blocking N-cadherin with specific antibodies in vitro inhibited the aggregation of isolated follicular cells and induced the apoptosis of mouse and human follicular cells [83,84].

The N-cadherin protein is present at the surface of follicular cells facing each other and also facing the oocytes. This indicates that the adhesion between follicular cells occurs through the homotypic N-cadherin–N-cadherin interactions, while the adhesion between follicular cells and oocytes through heterotypic N-cadherin–E-cadherin interactions. The interactions between cadherins are calcium-dependent. The ovarian follicles cultured in a calcium-free medium lose the membrane-bound E- and N-cadherin and contact between follicular cells and follicular cells and oocyte. Other cell adhesion molecules, such as calcium-independent nectin, are unable to restore ovarian follicle integrity [60]. This shows that cadherins are critical for the maintenance of the ovarian follicle integrity and folliculogenesis.

The expression of E- and N-cadherin changes during ovary luteinization (formation of the corpora lutea). During this process, N-cadherin expression decreases and E-cadherin expression increases in luteinizing granulosa, which possibly enhances cell adhesion, leading to the consolidation of the corpus luteum [59]. N-cadherin gradually decreases in the corpora lutea. Luteal cells in the corpus luteum show strong expression in the early-luteal and mid-luteal phase, and only weak expression in the late-luteal phase [83]. The expression of cadherins in the ovaries, similar to testes, is regulated by hormones. The neutralization of FSH in utero in hamster leads to the decrease of N-cadherin expression and the increase of E-cadherin expression [58].

The VE-cadherin (vascular endothelial cadherin, Cdh5) is another cadherin, which is involved in ovarian function. VE-cadherin regulates angiogenesis. This cadherin is expressed in the vasculature of the ovarian theca and corpora lutea, and the angiogenesis is critical for the formation of ovarian follicles and corpus luteum. The inhibition of blood vessel formation inhibits folliculogenesis and luteinization [85,86]. VE-cadherin is essential for the endothelial cell–endothelial cell homotypic interaction during the formation of blood vessels. Mice injected with the anti-VE-cadherin antibodies showed a diminished vasculature in the theca around ovarian follicles, lowered number of follicles, and lowered number of corpora lutea [85,86]. This indicates that the VE-cadherin-mediated angiogenesis in the theca, which encloses the ovarian follicles, is crucial for the development of ovarian follicles and corpora lutea.

## 9. Cadherins in Gametes and Fertilization

The spermatozoa (sperm), after being released in the testes, are transported to the epididymis, where they mature and are stored in a specific milieu in the lumen of the epididymis duct. This milieu is maintained by the blood–epididymis barrier (BEB). E-cadherin is a key protein in BEB. It promotes the adhesion of the epididymis epithelial cells [87,88]. The highest expression of E-cadherin was reported in the first and the second part of the epididymis (caput and corpus), while the third part (cauda) showed the lowest level of expression. The level of N- and P-cadherin in the epididymis is negligible.

After the sperm ejaculation to the female reproductive tract, it journeys to the cumulus–oocyte complex (COC). Only a part of the sperm deposited in the vagina reaches the oviductal isthmus. In the isthmus, the sperm joins the oviductal epithelium and awaits the ovulation [89]. The spermatozoa are trapped by the oviduct through the direct interaction between the sperm head plasma membrane at the acrosomal region and the surface of the ciliated oviductal epithelium. During the ovulation, the spermatozoa are released from the oviduct the oviductal ampulla to fertilize the oocyte. The release from the oviduct is necessary for sperm capacitation and renders them competent for fertilization. The oviductal epithelium expresses E- and N-cadherin in mice and humans [89,90], and these two cadherins are probably involved in sperm trapping in the oviduct; however, further studies are required to explain this mechanism. The E- and N-cadherins are also expressed in the endometrium. E-cadherin is expressed in endometrium throughout the menstrual cycle, and the expression of N-cadherin is the highest in a proliferative phase and the lowest in the secretory phase of the menstrual cycle [90]. How these two cadherins are involved in the regulation of the menstrual cycle also requires future studies.

The E-, N-, and P-cadherin are present at the surface of sperm and oocytes [91]. In bovine spermatozoa, E-cadherin is localized to the flagellum and the head (acrosomal and post-acrosomal region) with an especially high signal in the acrosomal region of the acrosome-intact spermatozoa. When the sperm detaches from the oviductal epithelium, E-cadherin disappears from the acrosomal region (outer membrane), and sperm gain the capacity to fertilize the oocyte. During the process of fertilization, after acrosomal exocytosis, the E-cadherin signal is the highest in the inner acrosomal membrane that directly contacts the cumulus–oocyte complex during fertilization. This indicates that E-cadherin participates in sperm capacitation and especially in the process of fertilization [92].

The N-cadherin is localized in the flagellum and the head (acrosomal and post-acrosomal region) of rat and human spermatozoa, and the acrosomal region of murine spermatozoa. After exocytosis of the acrosome, N-cadherin re-localizes to the equatorial segment of the head [91,93].

The presence of E- and N-cadherin at the surface of oocytes has been reported in mouse, rat, hamster, bovine, and human [91,93]. These cadherins were also detected in the follicular cells (cells of the cumulus surrounding the oocyte) [91]. The presence of E- and N-cadherin at the surfaces of the sperm and oocytes indicates that these cadherins may be involved in the adhesion of the gametes during fertilization. This was tested experimentally by gamete incubation in the presence of anti-E-cadherin or N-cadherin antibodies, followed by the heterologous sperm penetration assay (SPA), in which human spermatozoa and zona pellucida-free oocytes of hamster or mouse are used. Both antibodies inhibited human sperm penetration of hamster and mouse oocyte [91,93]. Similar results were reported for bovine and murine assays [91]. These experiments proved that both E- and N-cadherin participate in the fusion of sperm–zona pellucida and sperm–oocyte plasma membranes during fertilization.

## 10. Cadherins in *Drosophila* Gonad Development

Gonad development in *Drosophila* differs from that in the vertebrates. The earliest anlage of the gonad in *Drosophila* are two groups (one at each side of the embryo) of mesodermal cells called SGPs (somatic gonadal precursors) [94]. Clusters of SGPs become invaded by immigrating PGCs. Then, germ cells and SGPs undergo the gonad coalescence to form a rounded structure. Gonad coalescence involves movements of germ cells and SGPs, leading to the transformation of cell aggregate into a condensed gonad (compaction). The gonad coalescence involves two distinct processes: gonad compaction and the ensheathment of germ cells by SGPs [95]. The E-cadherin (called in *Drosophila* the DE-cadherin) is required for both processes. The germ cells and SGPs express E-cadherin. During gonad coalescence, the SGPs upregulate E-cadherin expression, which enhances their affinity and simultaneously decreases the affinity to the surrounding mesoderm. This leads to the condensation of SGPs and their separation from a surrounding mesoderm. The upregulation of E-cadherin also enhances their adhesion with the oocytes, leading to the gonad compaction and the ensheathment by SGPs. The embryos with a mutation in E-cadherin undergo the initiation of the gonad compaction; however, this process is not completed, and the SGPs form an elongated band of loosely associated cells. The ensheathment of the oocytes by SGPs is also disrupted [95].

The ectopic overexpression of E-cadherin also leads to defects in *Drosophila* gonadogenesis [95]. The ectopic expression of E-cadherin in the mesoderm causes a dramatic decrease in the number of SGPs, which indicates defects in SGP differentiation (specification) from the rest of the mesoderm. SPGs require a higher expression of E-cadherin to separate from the surrounding mesoderm. The ectopic overexpression of E-cadherin in the germ cells disrupts ensheathment by SGPs. The germ cells form a tight cluster lacking the SGPs. Thus, a balance of E-cadherin expression appeared critical for the development of proper gonad architecture: both gonad compaction and germ cell ensheathment.

**Figure 4:**
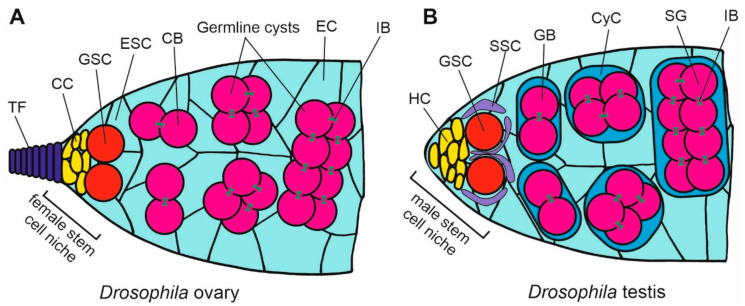
Scheme of organization of the stem cell niche in *Drosophila* ovary and testis. (**A**) In the ovary germarium, the female stem cell niche at the apical tip of the ovariole is maintained by the signaling from the somatic cap cells (CC), which are located below the terminal filament (TF), and bind, through E-cadherin, the germline stem cells (GSC). The divisions of GSCs give rise to the cystoblasts (CB) that develop into germline cysts that are surrounded by the escort cells (EC) originating from the escort stem cells (ESCs). (**B**) The apical tip of testis contains the male stem cell niche in which the somatic hub cells (HC) bind germline stem cells (GSC) through E-cadherin. GSCs are enclosed by the somatic stem cells (SSCs). Dividing GSCs give rise to the cysts of gonialblasts (GB) that differentiate into spermatogonia (SG) enclosed by the cyst cells (CyC). The germ cells in the cysts are interconnected by the intercellular bridges (IB) containing the ring canals.

E-cadherin is also crucial for the maintenance of the self-renewal niche at the apical tip of the *Drosophila* female and male gonads. Adult *Drosophila* ovaries are composed of several ovarioles. The apical end of each ovariole contains a specific niche of stem cells called the germarium. A tip of germarium is formed by cup cells to which germline stem cells (GSCs) are anchored. The GSCs give rise to the cystoblasts that proliferate and differentiate into oogonia. The somatic escort cells enclose the dividing germline cells (Figure 4A). E-cadherin is required for anchoring GSCs to the cap cells at the tip of the ovariole [56]. The deletion of E-cadherin from GSCs leads to the loss of stem cells [56]. Since the E-cadherin is also present between somatic cells, it is also required for maintaining the escort stem cells (ESCs) in their niche [55]. E-cadherin prevents stem cells from moving from the niche and thus from their differentiation. E-cadherin is also important for the proper arrangement of follicle cells around the oocyte in *Drosophila* egg [96].

The apical-end niche in the *Drosophila* testis maintains the continuity of spermatogenesis (Figure 4B). The tip contains a cluster of hub cells to which GSCs are anchored by E-cadherin [55]. GSCs are partially surrounded by the somatic stem cells (SSCs). The division of GSCs give rise to the gonialbalsts that differentiate into spermatogonia enclosed by cyst cells. E-cadherin is probably also crucial for the formation of the niche during the gonad development, while a higher signal of E-cadherin is visible at the anterior end of the differentiating *Drosophila* testis, where the hub is being established [95]. The N-cadherin is another adhesion molecule involved in the anchoring of GSCs in the testis niche in *Drosophila*. An overexpression of N-cadherin leads to the increased accumulation of stem cells in the niche [97].

In the germ cyst, the germ cells are connected by the intercellular bridges that are the result of the incomplete divisions. The bridges are maintained by the ring canals (RCs) that are protein complexes assembled inside the bridge. It has been shown that in *Drosophila*, the AP1 (clathrin adaptor protein 1) is responsible for the anchoring of the ring canal to the plasma membrane [98]. The ring canals are surrounded by the clusters of E-cadherin. The localization of E-cadherin in this position is regulated by AP1. E-cadherin is required for the maintenance of the RCs’ anchorage [98]. This indicates that E-cadherin is important for the maintenance of germ cell cyst structure.

The border and nurse cells isolated from *Drosophila* ovaries were used as an in vitro model to study the role of E-cadherin in cell adhesion and migration [99]. Border cells coalesce and move as a group in between nurse cells. E-cadherin at the leading edge of a migrating cell, acting through the small GTPase Rac, participates in the direction-sensing mechanism. This indicates that E-cadherin is involved in the mechanism orchestrating chemotaxis in vivo in insect cells.

Among insects, the honey bee is another species in which the localization of E- and N-cadherin was studied [100]. These two cadherins are present in the cytoplasm and in the nuclei of germ and somatic cells in the male and female gonads. In the testes, E-cadherin is present in the fusomes (i.e., membrane vesicles originating from endoplasmic reticulum and located in the ring canal), indicating its role in the cyst organization. In ovaries, E- and N-cadherin are present in the cortex of late oocytes, indicating the role of these two cadherins in the adhesion of honey bee ovarian cells.

## 11. Conclusions

The development of the germ cells and gametes depends on the contact with the surrounding somatic cells. Among a plethora of cell adhesion molecules expressed in the gonadal cells, only the role of E- and N-cadherin has been well characterized. These two cadherins participate at different steps of gonad development and gametogenesis. They assure adhesion between adjacent cells, regulate cell migration, cell sorting, segregation from the surrounding tissues, cell differentiation, and the control size of a given cell pool by the regulation of cell apoptosis and survival. Thus, these cadherins play not only the structural functions of guaranteeing tissue integrity but also the regulatory functions. However, because we still know very little about the role of other cadherins and other cell adhesion molecules in gonad development, further studies of gonad development and gonadogenesis are necessary.

The novel methods of molecular analyses should allow finding the answer to the following questions:

1. Which molecular mechanisms hide behind the requirement of cadherins, and other cell adhesion molecules (CAMs), for germ cells survival, and how do the cadherins prevent apoptosis of these cells?
2. Is the properly executed adhesion between germ cells and somatic cells sufficient to prevent their apoptosis, or are the more complex cadherin-related signaling pathways involved?

In addition, understanding the role of cadherins in the building and maintaining tissue structure of the developing gonads requires additional molecular analyses. Gonads are formed by complex rearrangements of cells of different origins, and the dialogue between them is probably critical for developmental success. However, we still are unable to define the particulars of this dialog despite that we know that cadherins, and the cell adhesion molecules in general, play a major role in this process.

## Figures and Tables

**Table 1:** Classification of cadherin superfamily according to [8].

Family	Type	Examples
**Classical cadherins (CDH)**—contain at least five conservative cadherin repeats in ectodomain, bind actin microfilaments via catenins	**Type I classical cadherins**—contain HAV (His-Ala-Val) motif in the first extracellular repeat	5 cadherins: E-, N-, P- R-, M-cadherin (CDH1, 2, 3, 4, 15)
	**Type II classical cadherins**—contain no HAV motif in the first extracellular repeat	13 cadherins including e.g., VE-cadherin (CDH5, 6, 7, 8, 9, 10, 11, 12, 18, 19, 20, 22, 24)
	**7D cadherins**—contain seven extracellular cadherin repeats and only a portion of the Ca^2+^-binding motif at the interface between cadherin repeats 2 and 3	2 cadherins: CDH16, CDH17
	**Desmosomal cadherins**—act as transmembrane proteins in desmosomes and bind intermediate filaments via desmoplakin	3 desmocollins (DSC1-3) and 4 desmogleins (DSG1-4)
	**CELSR cadherins** —Cadherin EGF LAG seven-pass G-type receptors	3 receptors: CELSR1,2,3
**Protocadherins (PCDH)**—contain ectodomain comprising six or seven cadherin repeats, transmembrane domain, and distinct, protocadherin-specific cytoplasmic domain	**Clustered protocadherins**	12 PCDHs, such as PCDH1, PCDH7
	**Non-clustered protocadherins**	64 PCDHs, such as PCDHA1, PCDHB1
**Related cadherins (CDHR)**—comprise at least two consecutive typical cadherin motifs	—	17 cadherins, such as CDHR1, FAT1-4, calsyntenins CLSTN1-3, RET

**Table 2:** Expression of cadherins in sexually differentiating developing mouse gonads (between E11.0 and E13.8) (according to [33]). Expression was studied in XX and XY supporting cells, XX stromal cell, XY interstitial cells, and XX and XY germ cells isolated from the gonads.

CadherinType	Gene Symbol	Site of Expression
Type I cadherins	*Cdh1* (E-cadherin)	germ cells in XX and XY gonads
	*Cdh2* (N-cadherin)	high expression in XX and XY supporting cells, lower in interstitial/stromal cells
	*Cdh3* (P-cadherin)	expression in all gonadal cells, higher in XY supporting and XY germ cells
Type II cadherins	*Cdh5* (VE-cadherin)	interstitial/stromal cells
	*Cdh6* (K-cadherin)	interstitial/stromal cells
	*Cdh9* (T1-cadherin)	XX and XY supporting cells
	*Cdh10* (T2-cadherin)	interstitial/stromal cells
	*Cdh11* (OB-cadherin)	high expression in interstitial/stromal cell, lower in supporting cells
	*Cdh12* (N2-cadherin)	residual expression
	*Cdh13*	residual expression
	*Cdh18*	high expression in all gonadal cells
	*Cdh23*	residual expression, higher in XY supporting cells
	*Cdh24*	residual expression
Desmosomal cadherins	*Dsg2* (desmoglein 2)	supporting and germ cells
	*Dsc2* (desmocollin 2)	somatic gonadal cells, higher in supporting than interstitial/stromal cells
Clustered protocadherins	*Pcdh1*	residual expression
	*Pcdh7*	residual expression
	*Pcdh9*	germ cells
	*Pcdh11x*	all studied gonadal somatic cells
	*Pcdh12*	residual expression
	*Pcdh17*	residual expression
	*Pcdh18*	high expression in gonadal somatic cells
	*Pcdh19*	residual expression
Non-clustered protocadherins	*Pcdhb3*	residual expression
	*Pcdhb16*	residual expression
	*Pcdhb17*	interstitial/stromal cells
	*Pcdhb18*	residual expression
	*Pcdhb19*	interstitial/stromal cells
	*Pcdhb20*	interstitial/stromal cells
	*Pcdhb21*	residual expression
	*Pcdhb22*	residual expression
	*Pcdhga12*	residual expression
Other cadherins	*Celsr1*	residual expression
	*Clstn1*	all studied gonadal cells
	*Dchs1* (Dachsous)	interstitial/stromal cells
	*Fat1*	all studied gonadal cells, higher in XY supporting cells, lower in XX supporting cells
	*Fat3*	interstitial/stromal cells
	*Fat4*	somatic gonadal cells, higher in XX supporting cells
